# Danshen (*Salvia miltiorrhiza*) Injection Suppresses Kidney Injury Induced by Iron Overload in Mice

**DOI:** 10.1371/journal.pone.0074318

**Published:** 2013-09-16

**Authors:** Shengjiang Guan, Juanjuan Ma, Ying Zhang, Yonggang Gao, Yuanyuan Zhang, Xuan Zhang, Na Wang, Yun Xie, Jiangyan Wang, Jianping Zhang, Li Chu

**Affiliations:** 1 Department of Pharmacology, School of Basic Medicine, Hebei Medical University, Shijiazhuang, China; 2 Department of Biochemistry, Bethune Military Medical College, Shijiazhuang, China; IIT Research Institute, United States of America

## Abstract

**Objectives:**

Excessive iron can accumulate in the kidney and induce tissue damage. Danshen (*Salvia miltiorrhiza*) injection is a traditional Chinese medicinal preparation used for preventing and treating chronic renal failure. The aim of the present study was to evaluate the effects of treatment with Danshen injection on iron overload-induced kidney damage.

**Methods:**

Mice were mock-treated with saline (control group) or given a single dose of iron dextran without treatment (iron overload group, 50 mg/kg/day for 2 weeks) or with daily treatments of low-dose Danshen (3 g/kg/day), high-dose Danshen (6 g/kg/day) or deferoxamine (100 mg/kg/day).

**Results:**

Treatment of iron-overloaded mice with Danshen injection led to significant improvements of body weight and decreased iron levels in the kidney. Danshen injection treatment also reduced concentrations of blood urea nitrogen, creatinine and malondialdehyde and enhanced glutathione peroxidase and superoxide dismutase activities. Histopathological examinations showed that Danshen injection ameliorated pathological changes and reduced iron deposition in kidneys of iron overloaded mice. Furthermore, the treatment was demonstrated to suppress apoptosis in nephrocytes.

**Conclusions:**

These results indicated that Danshen injection exerted significant renal protective effects in iron-overloaded mice, which were closely associated with the decrease of iron deposition and suppression of lipid peroxidation and apoptosis in the kidney.

## Introduction

Iron plays an important role in many metabolic and biological processes as a crucial micronutrient of cells. However, excessive iron is toxic to the human body and can accumulate in a wide variety of tissues, including the liver, heart and kidney, leading to oxidative injury and functional renal abnormalities, such as renal toxicity [1] and renal carcinoma [2]. Iron overload in the body occurs under conditions that create an imbalance in iron metabolism, such as hereditary hemochromatosis or hemosiderosis resulting from circumstances that bypass normal iron homeostasis (e.g., repeated blood transfusions, acute or chronic iron poisoning) [3]. Currently, iron chelators, such as deferoxamine (DFO) and deferiprone (DFP), are widely used to treat iron overload diseases [4]. Despite the therapeutic effectiveness of these drugs, some disadvantages exist, for example, hepatic dysfunction, allergy, agranulocytosis and poor patient compliance [5], [6]. Therefore, development of new treatments for iron overload diseases would be highly beneficial. In recent years, L-type calcium channels (LTCC) have been demonstrated to play an important role in the transportation of iron in excitable cells [7], [8]. Excess iron in the circulation above the binding capacity of serum transferrin (i.e., nontransferrin-bound iron) is released into the circulation and enters cells in the ferrous form through LTCC [9]. The result is the formation of highly reactive oxygen free radicals and oxidative damage to cellular proteins [10]. Consequently, calcium channel blockers have become potential new therapies for reducing oxidative injury induced by iron overload in these organs.

**Figure 1:**
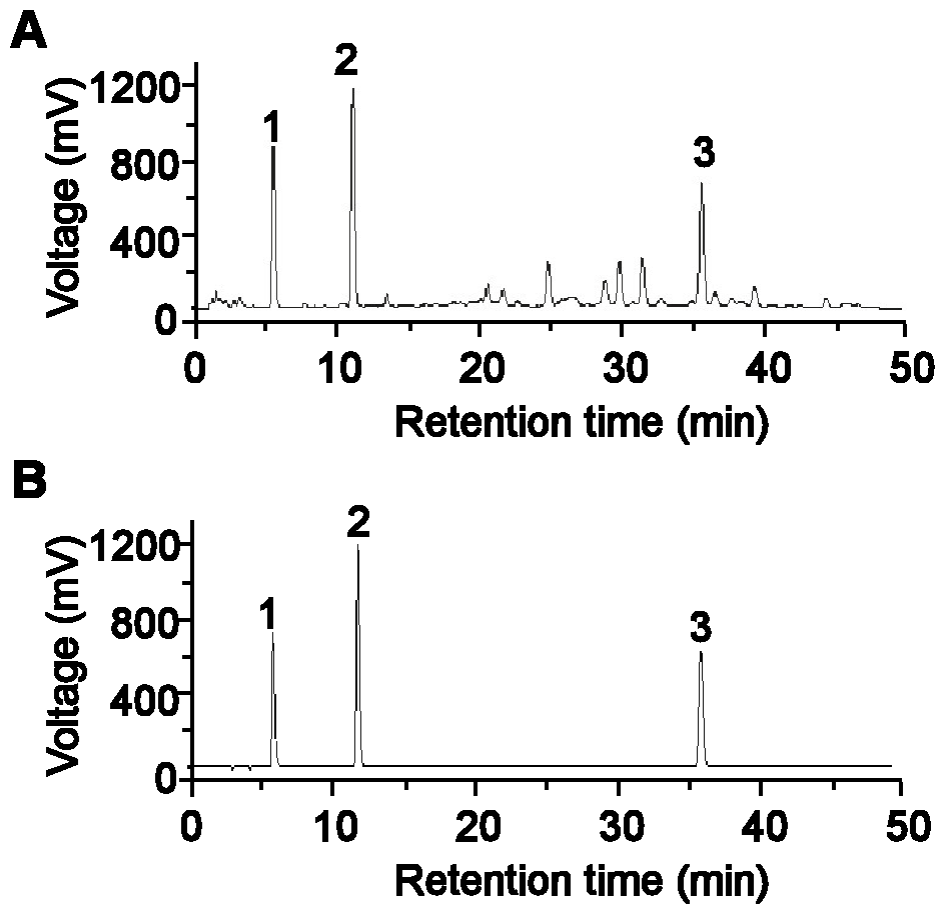
HPLC chromatographic profiles of major active components of Danshen injection. (A) Danshen injection. (B) Standards. Peaks represent: 1, 3,4-dihydroxyphenyl lactic acid; 2, protocatechuic aldehyde; 3, salvianolic acid B.

*Salvia miltiorrhiza*, commonly known as Danshen, is a traditional Chinese herbal medicine. Danshen injection made from the aqueous extracts of *S. miltiorrhiza* has been widely used to treat a variety of renal diseases, such as acute renal failure and acute nephritis, with positive effects in clinical practice [11]. Furthermore, the anti-oxidative and anti-inflammatory properties of Danshen as well as its protective effects on various organs have been investigated extensively [12]. Recent studies have indicated the diversity of the potential effects of Danshen in attenuating microcirculatory disturbances, including anti-oxidation [13], inhibition of apoptosis [14] and amelioration of injury to target organs such as the kidney [15]. The main active components of Danshen Injection, as analyzed by HPLC with UV detection, are 3,4-dihydroxyphenyl lactic acid (Danshensu), protocatechuic aldehyde and salvianolic acid B (Fig. 1). 3,4-dihydroxyphenyl lactic acid and salvianolic acid B exert anti-oxidative and hydroxyl radical scavenging activities. Protocatechuic aldehyde could suppress the migration and proliferation of vascular smooth muscle cells [12], [13]. The mechanism for some of its observed activities may be related to inhibition of LTCC [16] and prevention of Ca^2+^ overload [17]. In particular, our recent study demonstrated that Danshen injection could significantly prevent iron overload-induced damage in the mouse liver [18], [19].

Although previous studies strongly suggest that Danshen injection would be useful in treatment of iron overload diseases, its protective effects against kidney damage by excess iron have not been reported. Therefore, the purpose of this study was to investigate the effects of Danshen injection on nephrotoxicity induced by iron overload and the potential mechanisms in mice.

## Materials and Methods

### Materials

Danshen injection, prepared from the aqueous extracts of Danshen (dried root of *S. miltiorrhiza* belonging to the Labiatae family of flowering plants), was obtained from Shenlong Pharmaceutical Co., LTD. (Jiangsu, China). DFO and iron dextran injection were purchased from Novartis Pharma Stein AG (Stein, Switzerland) and Pharmacosmos A/S (Holbaek, Denmark), respectively. Unless otherwise stated, other chemical reagents were obtained from Sigma Chemical Co. (St. Louis, MO, USA).

### Animals

75 male Kunming mice weighing 20.0±2.0 g were provided by the Experimental Animal Center of Hebei Medical University. They were housed in a controlled environment and allowed free access to water and food. All animal handling procedures were in accordance with the Guidelines of Animal Experiments from the Committee of Medical Ethics, Ministry of Health of China, and experiments were approved by the Ethics Committee for Animal Experiments of Hebei Medical University (approval number: HEBMU -2010-10; approval date: October 25, 2010).

### High-performance Liquid Chromatography (HPLC) Analysis of Danshen Injection

Components of Danshen injection were analyzed by HPLC (Type L-6200A; Breeze work station at Hitachi Company, Japan). The mobile phase was a mixture of acetonitrile and 0.5% (v/v) orthophosphoric acid employing gradient elution (from 12∶88 to 35∶65, v/v) at a flow rate of 1 ml/min. The column was maintained at 30°C, and the detection wavelength was 288 nm. The results determined that the actual concentrations of the three major components of Danshan injection, 3,4-dihydroxyphenyl lactic acid, protocatechuic aldehyde and salvianolic acid B, to be 2.15, 0.44 and 1.01 mg/ml, respectively (Fig. 1).

### Experimental Treatments

After one week of acclimation, all mice were divided into the following five groups of 15 animals per group: control, iron overload, DFO, low-dose Danshen (L-Danshen) and high-dose Danshen (H-Danshen) groups. Mice of the latter four groups were intraperitoneally (i.p.) injected with a single dose of iron dextran 50 mg/kg/day, and the control group received an i.p. injection with isovolumic saline. Mice of the L-Danshen and H-Danshen groups were given i.p. injections of Danshen (3 and 6 g/kg/day, respectively) at 4 h before the administration of iron dextran on the same day, while mice of the control group and iron overload group received isovolumic saline by the same route. Mice in the DFO group were likewise injected i.p. at the dose of 100 mg/kg/day at 4 h before the administration of iron dextran on the same day.

On the 14th day, the final body weight of each animal was recorded, and overnight fasted animals were anesthetized with sodium pentobarbital (50 mg/kg). Blood from each animal was collected, and the serum was separated for further analysis. The kidney was quickly excised and washed in ice-cold phosphate buffer solution (PBS) and frozen in liquid nitrogen.

### Histological Examination of Kidney Tissues

Kidney tissues were fixed with 4% buffered paraformaldehyde, hydrated in ascending grades of ethanol, cleared in xylene and embedded in paraffin. Tissue sections (4 µm thickness) were cut and prepared for Prussian blue, hematoxylin and eosin (H&E), periodic acid–Schiff (PAS) and Masson’s trichrome staining, according to the manufacturer’s protocol. Glomerular damage was assessed by light microscopy and scored as described elsewhere [20]. Briefly, the score of each of three types of glomerular lesions, including glomerular hypercellularity, glomerular segmental lesions (crescents, adhesions and segmental sclerosis) and global glomerular sclerosis, was determined as follows (combined possible score range: 0–12): 0, no lesion; 1, lesion in <10% of glomeruli; 2, lesion in >10% but <25% of glomeruli; 3, lesion in >25% and <50% of glomeruli; 4, lesion in >50% of glomeruli. Tubulointerstitial damage was graded according to a previous study [21] on a scale of 0–4 as follows: 0, normal; 0.5, small focal areas; 1, involvement of <10% of the cortices and outer medullae; 2, 10–25% involvement of the cortices and outer medullae; 3, 25–75% involvement of the cortices and outer medullae; 4, extensive damage involving more than 75% of the cortices and outer medullae. All analyses were performed in a blinded manner.

### Terminal Deoxynucleotidyl Transferase Mediated dUTP Nick end Labeling (TUNEL) Assay

TUNEL staining was performed using an In Situ Cell Death Detection Kit (Roche, Mannheim, Germany) according to the manufacturer’s protocol. Briefly, the sections were deparaffinized, hydrated in a successive series of alcohol, washed in water followed by PBS and deproteinized by proteinase K. Thereafter, the sections were rinsed and incubated in the TUNEL reaction mixture. After additional rinses, sections were visualized using converter-POD, which is a peroxidase (POD)-conjugated anti-fluorescein antibody, with 0.02% 3,3′-diaminobenzidine (DAB), and then counterstained with hematoxylin. Number of tubular cells undergoing apoptosis was counted in three kidney sections, and the average number of TUNEL-positive cells/mm^2^ was calculated for each animal. All counting procedures were performed in a blinded fashion.

### Measurement of Total Iron in Kidney Homogenate

Total iron levels in kidney tissue were measured by flame atomic absorption spectroscopy (FAAS) [22]. To prepare frozen kidney homogenates for FAAS, the samples were dried at 65°C, weighed and then ashed at 500°C. Ashed samples were digested in hydrochloric acid and nitric acid before boiling in hydrogen peroxide until only 0.5 mL of liquid remained. Absorbance readings were performed at 248.3 nm using the Varian Spectra AA-10 Spectrophotometer (Agilent Technologies, Santa Clara, CA, USA). Standard curves for iron were prepared from commercially available standards.

### Assessment of Biochemical Indices in Serum

The level of blood urea nitrogen (BUN) was measured with the BUN kit (Jian Cheng Biological Engineering Institute, Nanjing, China) based on the Fearon reaction. Briefly, under the acidic condition with heating, urea nitrogen and diacetyldioxime reactions generate a red condensation product, the absorbance values of which can be obtained at the wavelength at 520 nm to determine the BUN level.

The total serum creatinine (CR) activity was detected in the serum using a CR kit (Jian Cheng Biological Engineering Institute). The principle of this assay kit is based on the Jaffe reaction where serum CR, in the presence of picric acid, generates the jacinth compound that can be measured at the wavelength of 510 nm. All procedures were performed according to the manufacturer’s protocols.

### Detection of Glutathione Peroxidase (GSH-Px) and Superoxide Dismutase (SOD) Activities and Malondialdehyde (MDA) Content in Kidney Homogenate

Each kidney tissue was weighed to prepare a 10% (w/v) buffered homogenate. The homogenate was centrifuged, and the supernatant was used for biochemical analyses. The protein concentration of the supernatant was determined by the Lowry method using bovine serum albumin (BSA) as a standard. SOD activity in the homogenate was assessed by using a commercially available kit (Jian Cheng Biological Engineering Institute), which is based on the auto-oxidation of hydroxylamine. The developed blue color of the reaction was measured at 550 nm.

Activity of GSH-Px was determined by the velocity method using a GSH-Px kit (Jian Cheng Biological Engineering Institute). The reaction is initiated by the addition of H_2_O_2_. A series of enzymatic reactions is activated by GSH-Px in the homogenate that subsequently leads to the conversion of GSH (reduced glutathione) to GSSG (oxidized glutathione). The change of absorbance during the conversion of GSH to GSSG can be recorded spectrophotometrically at 412 nm.

The MDA concentration in the homogenate was determined using a commercially available kit (Jian Cheng Biological Engineering Institute) based on thiobarbituric acid (TBA) reactivity. Briefly, after mixing trichloracetic acid with the homogenate and centrifugation, the supernatant was taken, and TBA was added. The developed red color of the resulting reaction was measured at 532 nm with a spectrophotometer. Other conditions and procedures were carried out as outlined by the manufacturer’s protocols.

### Statistical Analysis

Data were expressed as means ± S.E.M. All data were analyzed statistically using one-way analysis of variance (ANOVA) followed by Student’s *t*-test. Statistical significance was considered at *P*<0.05.

## Results

### Effects of Danshen Injection on Body Weight

**Figure 2:**
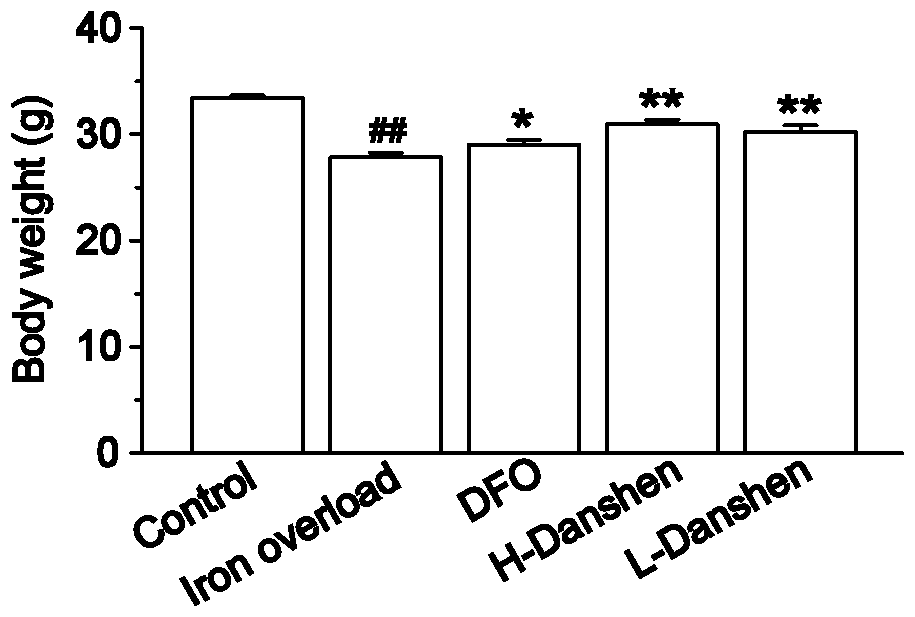
Effects of Danshen injection on body weight. Body weights of mice in the control, iron overload, DFO, H-Danshen and L-Danshen groups observed at day 14 of the experiment. Data are presented as means ± S.E.M of 15 animals per group. *P<0.05, **P<0.01, compared with iron overload group; *^##^P*<0.01, compared with control group.

Body weights of mice were compared in the different groups after two weeks (Fig. 2). Iron overload caused a significant decrease in weight gain, compared with the control group (*P*<0.01). Meanwhile, Danshen and DFO treatment markedly enhanced the weight gain, compared with the iron overload group (*P*<0.01 and *P*<0.05, respectively).

### Effects of Danshen Injection on Iron Deposition in Kidneys Observed by Prussian Blue Staining

**Figure 3:**
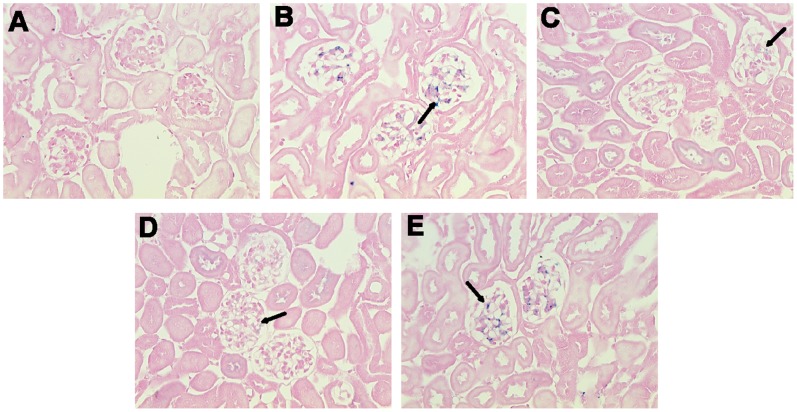
Effects of Danshen injection on iron deposition in kidneys. Representative microscopic photographs of kidneys stained with Prussian blue (magnification 400×). Samples were obtained from the control group (A), iron overload (B), DFO (C), H-Danshen (D) and L-Danshen (E) groups. Black arrows indicate iron deposits in glomerular cells.

Large numbers of blue-stained iron deposits were seen in the glomeruli of kidney sections in the iron overload group (Fig. 3), compared with the control group. Meanwhile, levels of blue-stained iron deposits in the DFO and Danshen groups were significantly reduced compared with the iron overload group.

### Effects of Danshen Injection on Morphological Changes in Kidneys

**Figure 4:**
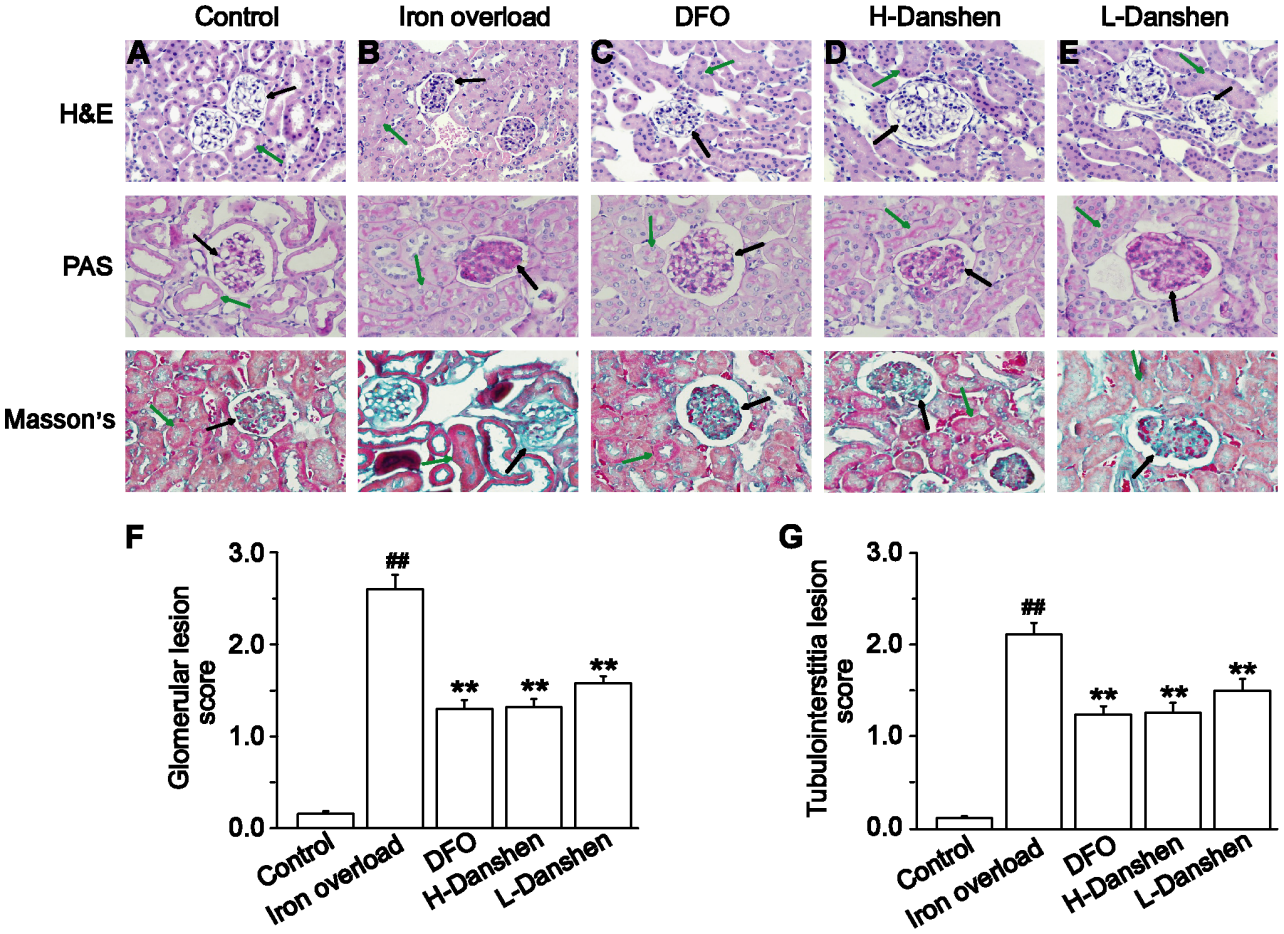
Effects of Danshen injection on morphological changes in kidneys. Representative microscopic photographs of H&E, PAS and Masson’s trichrome stained kidney tissue sections (magnification 400×). Kidneys were obtained from the control (A), iron overload (B), DFO (C), H-Danshen (D) and L-Danshen (E) groups. Scores for glomerular lesions (F) and tubulointerstitial lesions (G) are shown. Black arrows indicate histological changes of glomerular cells, and the green arrows indicate histological changes of tubulointerstitial cells. Data are presented as means ± S.E.M of 15 animals per group. **P<0.01, compared with iron overload group; *^##^P*<0.01, compared with control group.

Histological studies were performed to assess the injury in the mouse kidney after iron overload, as well as the effects of Danshen on tissue damage. The kidney sections of the control group appeared healthy with normal tissue structures (Fig. 4A). By contrast, the kidney sections of iron-overloaded mice displayed marked histological changes. The scores for glomerular and tubulointerstitial lesions showed an approximately 21-fold increase in the lesion areas compared with those in the control group (*P<*0.01) (Fig. 4F and G). After treatments of iron overload mice with low or high doses of Danshen injection, the lesion areas were decreased approximately by 40% and 50%, respectively, compared to the iron overload group (*P<*0.01) (Fig. 4D–G).

### Effects of Danshen Injection on Iron Level in Kidney Homogenate

**Figure 5:**
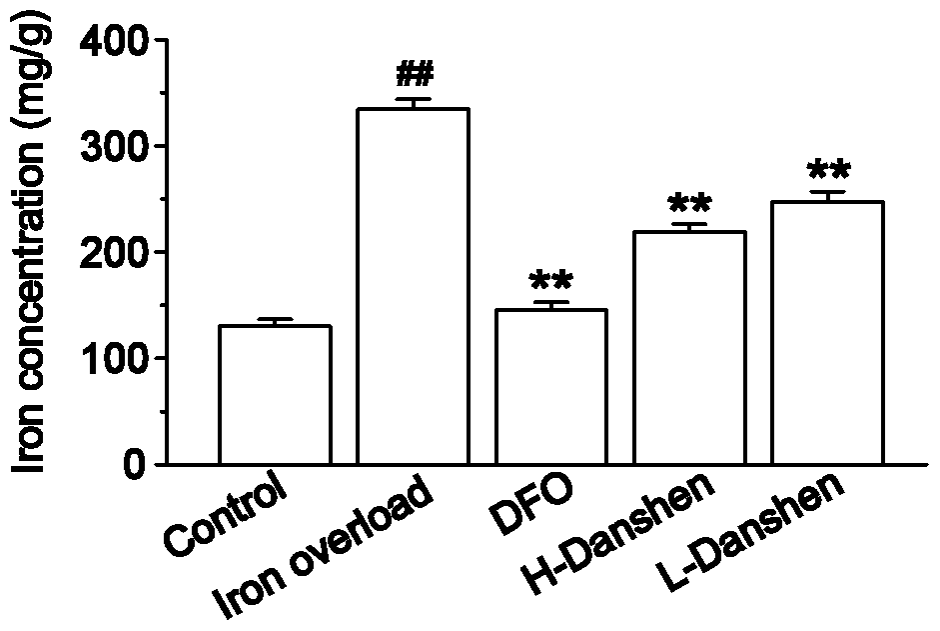
Effects of Danshen injection on iron levels in kidney homogenates. Iron levels were measured in kidney homogenates of control, iron overload, DFO, H-Danshen and L-Danshen groups. Data are presented as means ± S.E.M of 15 animals per group. **P<0.01, compared with iron overload group; *^##^P*<0.01, compared with control group.

As shown in Figure 5, iron dextran resulted in a dramatic elevation of total iron in kidney sections. Treatment with Danshen injection significantly decreased the level of iron (*P*<0.01), and this effect was dose-dependent.

### Effects of Danshen Injection on Levels of BUN and CR in Serum

**Figure 6:**
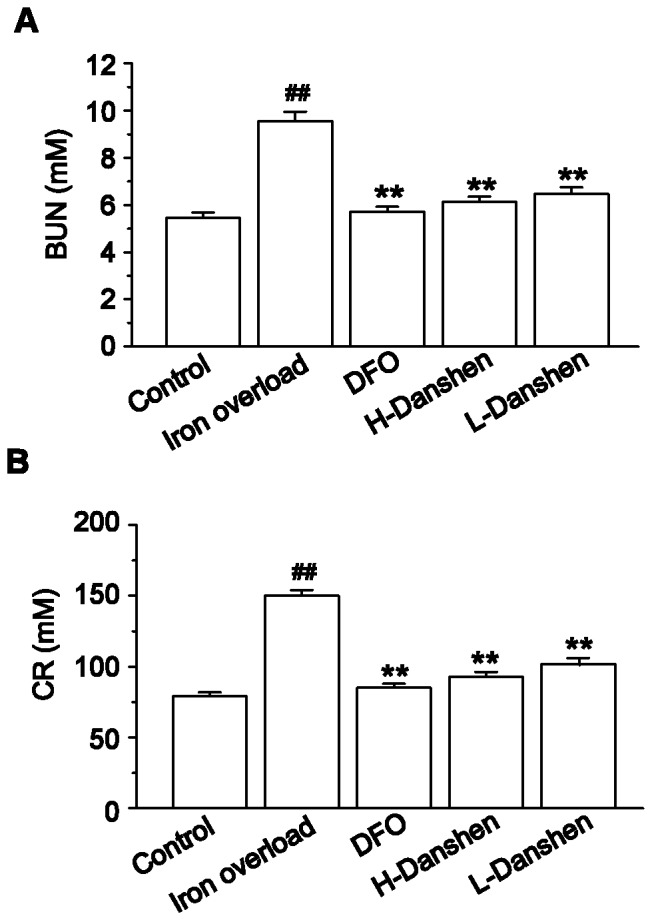
Effects of Danshen injection on serum BUN and CR levels. Serum BUN (A) and CR (B) levels were measured in control, iron overload, DFO, H-Danshen and L-Danshen groups. Data are presented as means ± S.E.M of 15 animals per group. **P<0.01, compared with iron overload group; *^##^P*<0.01, compared with control group.

Changes in serum BUN and CR levels in iron-overloaded mice with or without treatment are illustrated in Figure 6. Compared with control mice, BUN and CR levels were notably elevated in the iron-overloaded animals (*P*<0.01); however, these levels were clearly lower in the Danshen and DFO groups, compared with the iron overload group (*P*<0.01).

### Effects of Danshen Injection on SOD, GSH-Px and MDA in Kidney Homogenate

**Figure 7:**
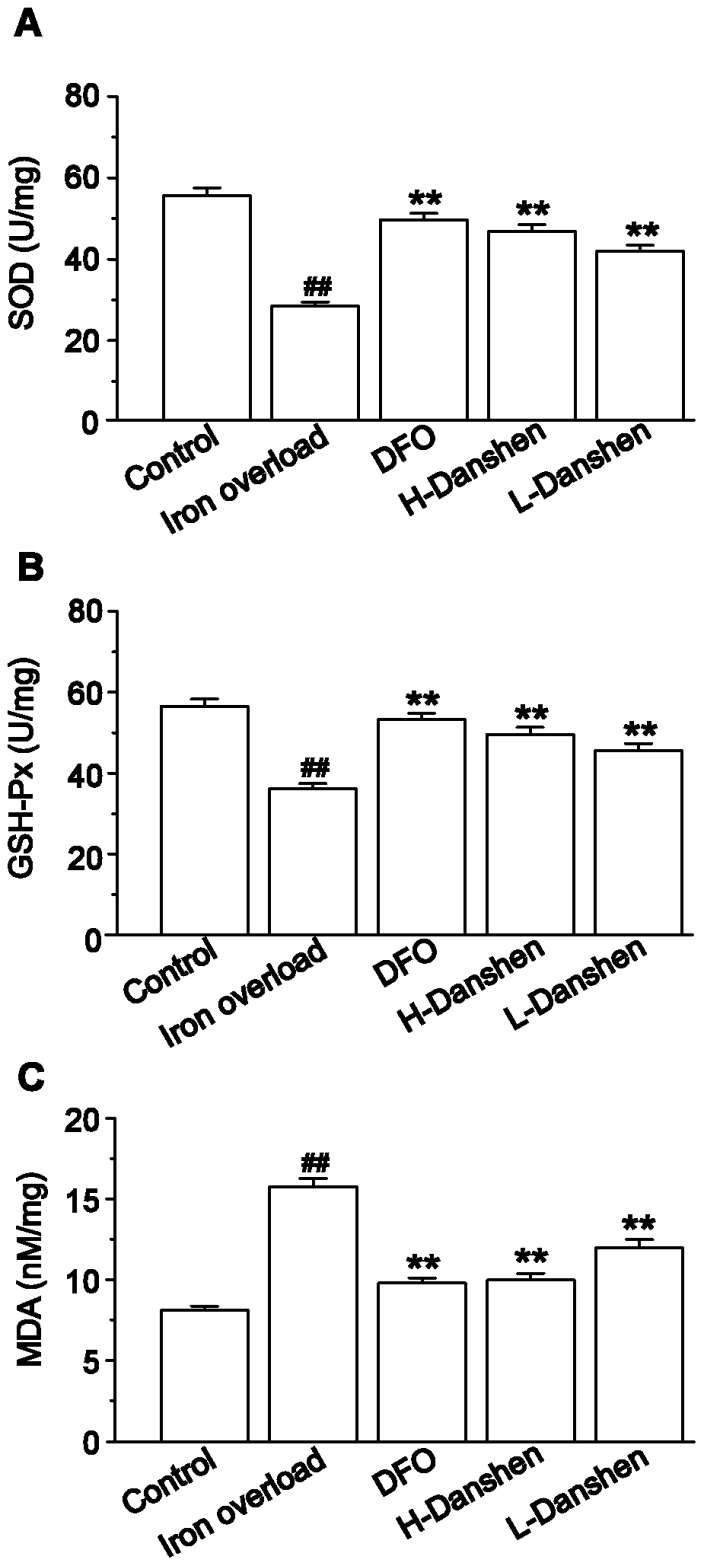
Effects of Danshen injection on concentration of MDA and activities of SOD and GSH-Px. SOD activity (A), GSH-Px activity (B) and MDA concentration (C) were measured in kidney homogenates of the control, iron overload, DFO, H-Danshen and L-Danshen groups. Data are presented as means ± S.E.M of 15 animals per group. **P<0.01, compared with iron overload group; *^##^P*<0.01, compared with control group.

Activities of SOD and GSH-Px as well as concentrations of MDA in kidney homogenates from the various mouse groups are shown in Figure 7A–C. Compared to those of the control group, SOD and GSH-Px activities were dramatically decreased in the iron overload group (*P*<0.01), while Danshen and DFO treated mice showed only a slight decrease in these activities (*P*<0.01) (Fig. 7A and B). Furthermore, the concentration of MDA was significantly enhanced in the iron overload group compared with the control group (*P*<0.01), while Danshen and DFO treatment markedly inhibited the excess iron-mediated enhancement of MDA levels (*P*<0.01) (Fig. 7C).

### Effects of Danshen Injection on Apoptosis in Kidneys

**Figure 8:**
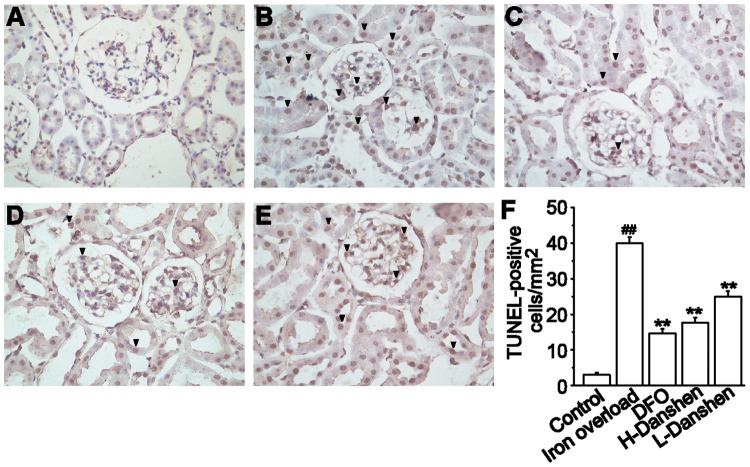
Effects of Danshen injection on apoptosis in kidneys. Representative microscopic photographs of TUNEL-stained kidney sections (black arrows) (magnification 400×). Kidneys were obtained from the control (A), iron overload (B), DFO (C), H-Danshen (D) and L-Danshen (E) groups. (F) Scores for TUNEL-positive cells in the kidneys are shown. Data are presented as means ± S.E.M of 15 animals per group. **P<0.01, compared with iron overload group; *^##^P*<0.01, compared with control group.

Apoptosis in tubular cells remained at a consistently low level in the control group (Fig. 8A and F), while TUNEL-positive tubular cells were significantly increased in the iron overload group (Fig. 8B and F, *P* *<*0.01). Danshen and DFO treatment dramatically decreased the extent of this apoptosis compared with the iron overload group (Fig. 8C–F, *P* *<*0.01).

## Discussion

Excess iron deposits in renal cells can lead to kidney oxidative injury and dysfunction [23]. Iron deposition in tissues of adult patients with β-thalassaemia hemoglobin E disease has been associated with abnormalities in renal function [24]. It is widely known that iron overload toxicity is related to free-radical-mediated tissue damage [25]. Officially listed in the Chinese Pharmacopeia, Danshen injection is widely used to treat coronary heart diseases, hemorrhage and renal diseases [11], [26]. Danshen can scavenge oxygen free radicals and inhibit lipid peroxidation [27]. Importantly, injectable Danshen has been used in clinical applications for more than 40 years with no serious adverse reactions reported [28]. Thus, we hypothesized that Danshen injection can protect renal tissue from damage induced by iron overload.

In the present study, we chose to construct an iron overload mouse model by administering an i.p. injection of iron dextran as reported previously [18]. The serum levels of BUN and CR can be used as an index for renal dysfunction and damage. Our results revealed that the levels of both BUN and CR in the serum were dramatically increased (Fig. 6), while the body weights significantly decreased (Fig. 2), compared with the control group at the end of the experimental period. Iron concentrations in the kidney homogenate of the iron overload group were significantly higher (*P*<0.01) than those in the control group (Fig. 5). Meanwhile the kidney sections of iron-overloaded mice displayed marked histological changes, and the scores for glomerular and tubulointerstitial lesions revealed an approximate 21-fold increase of the lesion area compared with that in the control group (*P<*0.01) (Fig. 4). Thus, iron dextran treatment in this mouse model was considered appropriate for studying kidney damage related to iron overload.

The marked deposition of iron in the kidney can injure renal cells and disturb kidney function, ultimately leading to reduced body growth [23]. In this study, our results showed that iron-overloaded kidney sections displayed marked deposition of iron, while both low and high doses of Danshen appeared to reduce iron deposits in the glomeruli as determined by microscopic observations of Prussian blue stained renal sections (Fig. 3). Additionally, Danshen treatments significantly decreased total iron levels in the kidney (*P*<0.01) compared with the iron-overloaded group at the end of the 14-day experiment as determined by FAAS (Fig. 5). LTCC are key transporters of iron into cardiomyocytes under iron-overloaded conditions, and treatment with LTCC blockers such as verapamil can lead to the inhibition of the LTCC current in cardiomyocytes [8]. 3,4-dihydroxyphenyl lactic acid, as one of the active and water-extractable components of Danshen, has been demonstrated to inhibit calcium channels in vascular smooth muscle cells [29]. This observation suggests that Danshen can prevent the entry of iron into kidney tissue via negative effects on calcium channels. In this study, Danshen injection significantly reduced the amount and altered the distribution of iron deposits in the kidney, consequently ameliorating histological changes and improving renal function, as demonstrated by the dramatically decreased levels of both BUN and CR in the serum (both *P*<0.01) compared with the iron-overloaded group. Therefore, Danshen likely prevents the entry of iron into the kidney, thereby protecting tissue damage induced by iron overload. Alternatively, Danshen may have a similar function to that of DFO by chelating and clearing free iron ion to reduce deposition in the kidneys. However, these hypotheses will require further study to confirm.

Iron overload has been reported to lead to an increase in lipid peroxidation [30]. As a major product of lipid peroxidation, MDA can reflect indirectly the degree of damage to cells [31]. In our study, we observed that the concentration of MDA was markedly increased (*P*<0.01) in iron-overloaded mice (Fig. 7C), thus suggesting the induction of renal lipid peroxidation. SOD and GSH-Px are potent enzymatic antioxidants that scavenge harmful reactive oxygen species, bringing the first line of defense against free radicals by converting toxic superoxide into the less toxic hydrogen peroxide [32]. While prior studies have reported on the antioxidant activities of Danshen by scavenging free radicals [33], [34], our results provided convincing evidence that it could exert potent protective effects against lipid peroxidation induced by iron overload. At the dose of iron dextran given, the antioxidant defensive system in the kidney was insufficient to give complete protection against the induced free radical damage. The response of the kidney to the toxic iron was an escalation in the lipid peroxidation, accompanied by the increase of MDA concentration as well as decrease of SOD and GSH-Px activities. Meanwhile, Danshen treatment could increase the renal antioxidation apparently by restraining the content of MDA and simultaneously enhancing the activities of GSH-Px and SOD (Fig. 7), thus decreasing lipid peroxidation. The resulting balance in lipid peroxidation in the kidney then allowed the antioxidant defensive system of the kidney to effectively protect the organ from tissue damage due to iron overload-induced free radicals. This effect on antioxidants may be an important mechanism involved in the protective effects of Danshen injection against renal injury.

Apoptosis is a tightly controlled process in which cell death is executed for maintaining physiological homeostasis and for responding to varying noxious stimuli or in disease. Here we examined nephrocytes by TUNEL staining to investigate whether Danshen is associated with excess iron-induced apoptosis. Our findings revealed that the extent of TUNEL-positive staining in the kidney was dramatically increased in the iron overload group compared with the control group (*P<*0.01) (Fig. 8), while treatment with Danshen injection significantly decreased the apoptosis induced by excess iron in the kidney (*P<*0.01). Thus, the potential anti-apoptotic effect of Danshen injection may be another mechanism for its attenuation of renal injury induced by iron overload.

## Conclusions

Our results preliminarily demonstrated that Danshen injection could significantly restore abnormal function and ameliorate the pathological changes of the kidney in iron-overloaded mice. These remarkable protective effects against renal injury caused by excess iron may be attributed to the prevention of iron deposition and inhibition of lipid peroxidation and nephrocyte apoptosis. While the precise protective mechanisms of Danshen injection warrant further studies due to its complex chemical composition and multiple pharmacological effects, our current findings may help to expand its clinical application for iron overload-induced nephrotoxicity.
